# Micrometric Wrinkled Patterns Spontaneously Formed on Hydrogel Thin Films via Argon Plasma Exposure

**DOI:** 10.3390/molecules24040751

**Published:** 2019-02-19

**Authors:** Carmen M. González-Henríquez, Diego F. Veliz-Silva, Mauricio A. Sarabia-Vallejos, Adolfo del Campo-García, Juan Rodríguez-Hernández

**Affiliations:** 1 Facultad de Ciencias Naturales, Matemáticas y del Medio Ambiente, Departamento de Química, Universidad Tecnológica Metropolitana, Las Palmeras 3360, Santiago 7800003, Chile; diegofvelizs@gmail.com; 2 Programa Institucional de Fomento a la Investigación, Desarrollo e Innovación, Universidad Tecnológica Metropolitana, Ignacio Valdivieso 2409, Santiago 8940577, Chile; 3 Escuela de Ingeniería, Departamento de Ingeniería Estructural y Geotecnia, Pontificia Universidad Católica de Chile, Avenida Vicuña Mackenna 4860, Santiago 7820436, Chile; masarabi@uc.cl; 4 Escuela de Ingeniería, Instituto de Ingeniería Biológica y Médica, Pontificia Universidad Católica de Chile, Avenida Vicuña Mackenna 4860, Santiago 7820436, Chile; 5 Ceramics for Smart Systems Group, Departamento de Electrocerámicos, Instituto de Cerámica y Vidrio- Consejo Superior de Investigaciones Científicas (ICV-CSIC), Kelsen 5, Madrid 28049, Spain; adelcampo@icv.csic.es; 6 Polymer Functionalization Group. Instituto de Ciencia y Tecnología de Polímeros-Consejo Superior de Investigaciones Científicas (ICTP-CSIC), Departamento de Química Macromolecular Aplicada, Juan de la Cierva 3, Madrid 28006, Spain; jrodriguez@ictp.csic.es

**Keywords:** wrinkled hydrogel surfaces, argon plasma oxidation, doctor blade, adhesion enhancing

## Abstract

The generation of microstructured patterns on the surface of a specific polymeric material could radically improve their performance in a particular application. Most of the interactions with the environment occur at the material interface; therefore, increasing the exposed active surface considerably improves their range of application. In this article, a simple and reliable protocol to form spontaneous wrinkled patterns using a hydrogel layer is reported. For this purpose, we took advantage of the doctor blade technique in order to generate homogenous films over solid substrates with controlled thickness and large coverage. The hydrogel wrinkle formation involves a prepolymerization step which produces oligomers leading to a solution with increased viscosity, enough for doctor blade deposition. Subsequently, the material was exposed to vacuum and plasma to trigger wrinkled pattern formation. Finally, a UV-polymerization treatment was applied to fix the undulations on top. Interestingly, the experimental parameters allowed us to finely tune the wrinkle characteristics (period, amplitude, and orientation). For this study, two main aspects were explored. The first one is related to the role of the substrate functionalization on the wrinkle formation. The second study correlates the deswelling time and its relationship with the dimensions and distribution of the wrinkle pattern. In the first batch, four different 3-(trimethoxysilyl)propyl methacrylate (TSM) concentrations were used to functionalize the substrate in order to enhance the adhesion between hydrogel film and the substrate. The wrinkles formed were characterized in terms of wrinkle amplitude, wavelength, pattern roughness, and surface Young modulus, by using AFM in imaging and force spectroscopy modes. Moreover, the chemical composition of the hydrogel film cross-section and the effect of the plasma treatment were analyzed with confocal Raman spectroscopy. These results demonstrated that an oxidized layer was formed on top of the hydrogel films due to the exposure to an argon plasma.

## 1. Introduction

Over the last few years, material surface micro-modification—both physically and chemically—has become an important topic for researchers in the area of material science due to its interesting characteristics which may be useful in various application fields such as optoelectronic devices [1,2], smart fabrics [3], biocompatible surfaces for cell growth [4], cell contact screening platforms [5], mechanical sensors, energy storage, and chemical detectors [6], among others. Surface modifications (chemical or topographical) can be generated at different length scales on the material surface by varying the experimental conditions for their fabrication. Chemistry and surface microstructure play a fundamental role in the surface properties of the material and, for instance in bio-related applications, these two characteristics significantly alter the way biological media react [7]. In this context, surface properties have been reported to be critical in the development of novel biomaterials. Accordingly, it has been possible to fabricate selective biofouling surfaces which can stimulate cell growth while avoiding bacterial colonization, just by changing the scale of the micro-patterns present on the surface [8].

Different methodologies for surface micro-modification have been carried out in the past years, such as laser/electron-beam, micro- [9,10] or nano-lithography [11], magnetron sputtering [12], photolithography [13], or chemical etching [14]. These methods have the advantage of allowing high resolutions and control in surface patterning shapes and dimensions, but at the expense of long fabrication times and expensive manufacturing processes. In previous studies, it was reported that the morphologies and sizes of the patterns formed on top of the materials had an intimate relationship with the thickness of the film itself. In fact, Zhang et al. [15] demonstrated that the dynamics of ultra-thin molecular glass films were related to their thickness variation. They showed that below 30 nm, the film presented a liquid-like behavior, which generated morphological changes in the patterns, increasing their roughness together with the layer thickness. Recently, the research group of Chung et al. [16] studied the relaxation dynamics of thin polymer films below glass transition temperature via the compression of polystyrene films supported on polydimethylsiloxane substrates. They demonstrated that the relaxation time and the morphology dynamics of wrinkled films were strongly dependent upon the temperature of films which presented thicknesses below 100 nm.

In this study, we propose an alternative approach that takes advantage of surface instabilities to spontaneously generate randomly distributed micro-patterns at affordable prices and in much lower fabrication times than the techniques previously mentioned [17]. In addition, surface patterning based on instabilities does not require expensive equipment for modifying the surfaces. Moreover, these techniques allow users to control the dimensions and the distribution of these micro-patterns by varying the experimental conditions.

Among the methodologies based on surface instabilities, which include among others dewetting, breath figure formation, electrohydrodynamic patterning, thermal-gradient-induced surface structuration, and reaction–diffusion surface patterning, wrinkle formation is probably one of the most intensively explored approaches to produce micro- and nanometer size functionalized wrinkled surfaces due to their interesting applications for the elaboration of optical surfaces, as templates for microparticle arrays, the elaboration of flexible electronics, or the control of the adhesive properties of the surface [8,18].

Different alternatives have already been reported to form wrinkled surface patterns. However, most of the methodologies are based on the employment of an external stimulus (temperature, mechanical stretching, etc.) applied over the top layer, which has different mechanical properties (usually a rigid layer) from the material foundation (soft). Then, by removing the external stimulus, the formation of a micro-wrinkled pattern is triggered due to mechanical stress mismatch [19], which accommodates the stress by producing an out-of-plane deformation of the surface.

In this case, the rigid top layer was formed over a biocompatible hydrogel film via a controlled deswelling process under a vacuum atmosphere. Then, by exposing the dried surface to argon plasma, the wrinkled pattern was spontaneously formed on top in just half a minute [20,21]. Both processes were necessary to generate the wrinkled pattern. The pattern formation parameters, like plasma exposure and vacuum time, were continuously varied in order to find the most optimal conditions to generate a homogenous and scalable microtopography on the hydrogel film.

In parallel, we studied the adherence of the film with the substrate. Increasing the adherence is crucial for some applications in which the layer needs to remain anchored for long periods of time under stressful conditions [22,23]. Accordingly, we varied the functionalization parameters of the substrate and observed the effect that these modifications had on surface pattern morphology and on the bonding forces with the substrate.

Interestingly, the variation of deswelling time and substrate functionalization had a relevant effect on pattern dimensions, whereas argon plasma exposure time had none. On the other hand, plasma exposure generated chemical differences on the rigid top layer. It seems that this process incited the creation of free radicals on the surface, which in the presence of air, quickly oxidized, forming a thin rigid layer on top [24,25].

## 2. Results and Discussion

### 2.1. Functionalization Effect on Wrinkle Hydrogel Film Adherence

#### 2.1.1. Layer Thickness and Contact Angle

The first series of experiments were focussed on the analysis of the functionalization step’s role in the wrinkle formation. The substrate functionalization process was performed by following the procedure explained in the experimental part reported by Yuk et al. [26], but by varying the 3-(trimethoxysilyl) propyl methacrylate (TSM) solution concentration from 0.005% to 2% *v/v*. The hypothesis is that based on the fact that TSM molecules are gradually chemisorbed on the silicon substrate, the chemical composition of the SAMs can also be varied. The relationship between TSM concentration and monolayer thickness is mainly based on the kinetic molecular law which establishes that, according to the species concentration, the number of molecular shocks will vary [27,28]. In this case, the TSM solution concentration influenced the effective collisions between the hydroxylated surface obtained before piranha treatment and the methoxy groups belonged to the silane molecules. The interaction between these radical groups generated a covalent anchoring between the TSM molecules and the substrate surface. The thicknesses of the TSM layer were characterized by using an ellipsometry technique together with static water contact angles. A multi-angle laser ellipsometer model SE400Adv, from SENTECH Instrument GmbH (Berlin, Germany), was used to perform optical measurements with variable incidence angles from 30° to 90° in steps of 0.5°; the equipment possesses an attached motorized goniometer from Hüber Diffraktionstechnik GmbH & Co. (Rimsting, Germany) for control of the incidence angle variation. A stabilized He-Ne laser (λ = 633 nm) allowed us to obtain measurements to a precision of ± 0.1 Å in the thin film thickness. It is important to mention that the ellipsometry measurements were carried out over silicon waters (Si(100)) instead of glass substrates because clear materials are not recommended for ellipsometry due to light intensity lost via backside reflection processes. The TSM functionalization process was the same for silicon wafers as for the glass substrates.

Water contact angle measurements were performed using a Theta optical tensiometer (Attension, from Biolin Scientific, Gothenburg, Sweden), adding 4 µL of liquid phase over the solid sample.

**Figure 1:**
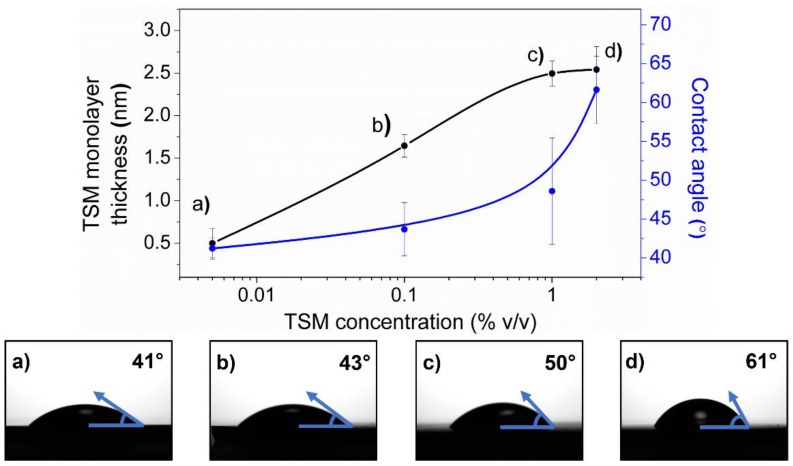
Above: Evolution of the thickness of the self-assembled 3-(trimethoxysilyl)propyl methacrylate (TSM) monolayer (black line) and static water contact angle (blue line) as a function of the TSM concentration employed ((**a**) 0.005%, (**b**) 0.1%, (**c**) 1%, and (**d**) 2% *v/v*). The data is plotted on a logarithmic scale. Below: Water contact angle images of the wrinkled surfaces; the right contact angle is drawn on each case.

As can be observed in Figure 1, the thickness of the TSM monolayer gradually increases with the concentration of the solution used in each case (black line). The thickness grows from ~0.5 nm to ~2.5 nm with the TSM concentration, which varies from 0.005% to 1% *v/v*. These results indicate that, at low concentrations (0.005% *v/v*), it is not possible to form a homogeneous grafted TSM layer on the silicon substrate surface. On the other hand, at 0.1% *v/v*, the TSM layer thickness increases substantially, agreeing with theoretical TSM molecular length [29,30], making it possible to declare that a TSM monolayer has been successfully grafted and self-assembled on top. Interestingly, after this point (1% and 2% *v/v*), the thickness of the layer tends to increase (~2.5 nm), entering into a plateau zone which is characteristic of a TSM bilayer formation. In short, after 1% *v/v*, the TSM deposition enters into a bilayer metastable regime [29].

Static water contact angles were also measured on the substrate surface (Figure 1, blue line). These values could serve as an indirect method to evaluate the amount of TSM molecules grafted onto the surface because the exposed vinyl groups of the methacrylate sector in their molecular structure have a characteristic hydrophobic nature [31]. Consequently, as the TSM molecules attach onto the surface, the substrate surface wettability decreases, increasing their contact angle. The results demonstrate that by increasing the TSM concentration, the static water contact angle grows, showing that substrate surface hydrophobicity increases with the TSM molecular amount present on the surface.

#### 2.1.2. TSM Concentration Effect on Wrinkled Pattern Morphology

Once the substrate silanization deposition was fully characterized, prepolymerized hydrogels were deposited on top of the silanized glasses, using the same vacuum exposure time (7 h) used previously. The films were obtained by using the same steps explained in the methodology section.

**Figure 2:**
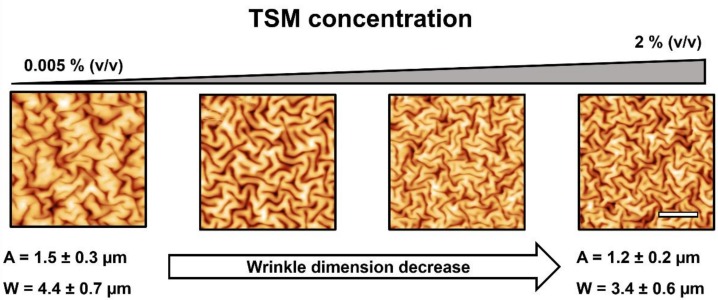
AFM micrographs of hydrogel films at different TSM concentrations (0.005%, 0.1%, 1%, and 2% *v/v*). A and W represent the mean amplitude and wavelength of the most extreme wrinkled patterns. The scale bar in the image corresponds to 10 µm.

Then, the wrinkled hydrogel films were characterized by using an AFM to measure the wrinkle characteristics. The results obtained are depicted in Figure 2; the wrinkle wavelengths and amplitudes according to TSM concentration are depicted in the Supplementary Materials section (Figure S1). In Figure 2, four different AFM micrographs are shown according to TSM concentration (from 0.005% to 2%). The wrinkle dimensions, amplitude (height), and wavelength (width) tend to slightly decrease with the TSM concentration increase from 1.5 ± 0.3 µm to 1.2 ± 0.2 µm in amplitude and from 4.4 ± 0.7 µm to 3.4 ± 0.6 µm in wavelength for the most extreme cases (TSM concentrations of 0.005% and 2%). This effect could be caused by the adherence variation between substrate and hydrogel film.

**Figure 3:**
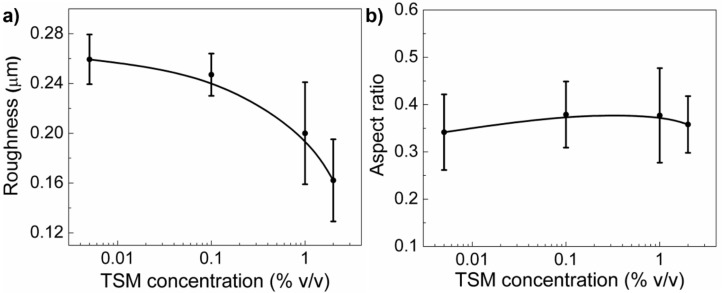
(**a**) Wrinkled pattern roughness and (**b**) aspect ratio of the hydrogel films deposited over silanized substrates at different TSM concentrations.

In Figure 3a–b, the roughness of the samples together with the aspect ratio of the wrinkles are depicted. In both cases, the TSM concentration used to silanized the substrate was varied from 0.005% to 2% *v/v*. As can be observed in Figure 3a, the roughness tends to decrease with TSM concentration, similarly with the amplitude and the wavelength of the wrinkles, as was expected (Figure S1), because smaller surface undulations produce a smaller roughness on top. Interestingly, in the case of the aspect ratio (Figure 3b), the tendency is different. In fact, the aspect ratio values almost do not change at all and remain close to ~0.35, which indicates that, independent of the TSM concentration used to silanize the substrate, the ratio between the amplitude and wavelength of the wrinkled patterns does not change, that is, although the dimensions of the wrinkled patterns (height and width) slightly decrease with the increase of TSM concentration, they do so together, thus keeping their variation ratio similar for all the cases.

In order to confirm the increasing adherence between the hydrogel layer and the substrate surface as a result of the increasing TSM concentration, cross-hatch adhesion tests were performed for each sample using a Cross-hatch adhesion test kit, model CC1000 from Dyne Testing Ltd. (Lichfield, UK), using a six-blade cutter spaced at 2 mm for coating thicknesses of no more than 60 µm, according to the standards ASTM D3359 [32] and ISO 2409 [33]. The kit uses an adhesive roll tape appropriate for soft coatings.

The results demonstrate that, at low TSM concentrations (0.005% *v/v* and 0.1% *v/v*), the adherence was rather poor resulting in almost no material attached after tape peeling (less than 10%). Wrinkled surfaces prepared using 1% *v/v* TSM concentrations presented improved adhesion and the remaining area after peeling increased up to 25 ± 2%. Finally, using a TSM concentration of 2% *v/v*, the area of material which remained attached to the surface increased up to 73 ± 3%, close to three times more than in the case of 1% *v/v*. These results are depicted in Table 1. The adhesion increase could be the reason for the wrinkle dimension decrease; as the adhesion with the substrate increases, the hydrogel film faces greater mechanical impediments, making it more difficult for the material to deform, thus altering the wrinkle dimensions. However, it is difficult to categorically make this statement because, at low concentrations, it becomes complicated to obtain homogenous TSM monolayers, thus producing a higher hydrogel film detachment from the substrate. Therefore, the synergy between these two effects could generate the wrinkle dimension variation on top of the film.

### 2.2. Effect of the Vacuum Exposure Time on the Wrinkle Dimensions

**Figure 4:**
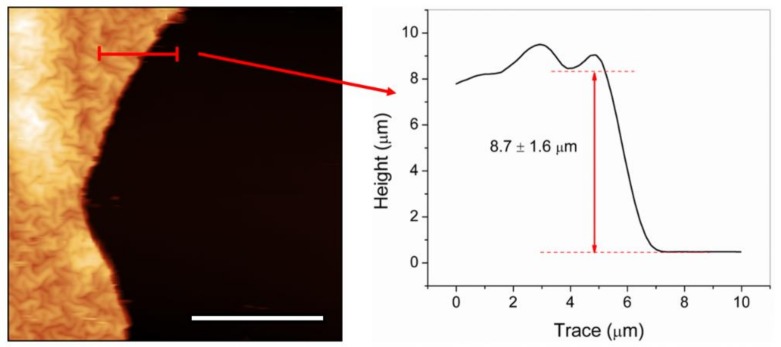
Cross-section profile obtained with AFM of a masked region from a sample deposited using the doctor blade method. The scale bar corresponds to 20 µm.

Once the prepolymerization step was achieved and the microgels were correctly deposited on top of the functionalized substrates using a concentration of 2% *v/v* of TSM (higher adhesion results), the retained water was eliminated from the film via a deswelling process under a rough vacuum atmosphere at room temperature (10^−2^ torr, 22 °C). As a result, the second important experimental parameter explored was the duration of the vacuum exposure. The vacuum exposure time was varied from 2 to 14 h. The hydrogel films obtained after the vacuum treatment were exposed to Ar^+^ plasma in order to trigger a polymerization on the deposited microgels [24,34]. The deswelling process is responsible for generating surface instabilities on the materials, which, upon polymerization/oxidation of the surface after Ar^+^ plasma etching, produce the spontaneous formation of wrinkled patterns on top due to a stress/strain mismatch between the top rigid layer and the soft foundation [20]. By using this strategy, it was possible to form homogeneously wrinkled and reproducible films of 8.7 ± 1.6 µm thickness, and this value was confirmed via AFM measurements in masked regions of the samples. An AFM micrography and a profile are depicted in Figure 4. Interestingly, the variation in TSM concentration, vacuum time, or plasma exposure time does not affect considerably the thickness of the hydrogel sample.

**Figure 5:**
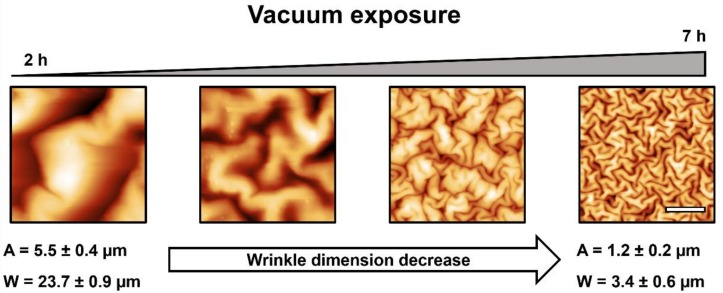
AFM micrographs of HEMA-PEGDA_575_ samples at different vacuum exposure times, from 2 h to 7 h. A and W represent the mean amplitude and wavelength of the most extreme wrinkled patterns. The scale bar corresponds to 10 µm.

Figure 5 shows the AFM micrographs of the micro-wrinkled patterns obtained via the variation of vacuum exposure time. Five micrographs are shown with an increasing deswelling degree (from 2 to 14 h of vacuum). As can be observed, the wrinkled pattern dimensions (wavelength and amplitude) tend to decrease with a vacuum exposure time increase, resulting in wavelengths of ~23.7 ± 0.9 µm for 2 h, to architectures with a wrinkle width of ~3.4 ± 0.6 µm for the samples fabricated with 7 h of vacuum; a similar situation occurs with wrinkle amplitude (from 5.5 ± 0.4 μm to 1.2 ± 0.2 μm). It is important to mention that at twice the time (14 h), flat surfaces were obtained instead of wrinkled patterns. This effect could be related to the stiffness increase of the polymer bulk with deswelling. The Young modulus of deswelled films was locally measured by using a force spectroscopy method and analyzed using the Hertz model for indentation with conical/pyramidal AFM tips over a soft flat surface [35,36]. The stiffness of the dried films varied from 0.6 ± 0.1 KPa in the case of 2 h deswelling time to 1.4 ± 0.2 KPa in the case of 7 h, data which are in concordance with the results obtained in González-Henríquez et al. [37]. As the water removed from the hydrogel increases with deswelling time, the film becomes more rigid and therefore generates a smaller wrinkle wavelength according to the equation obtained from the articles of Rodríguez-Hernández [19] and Nania et al. [21,34]:

(1) $$\lambda =A\;t\;{\left[\frac{E_{s}\;\left(1-v_{p}^{2}\right)}{E_{p}\;\left(1-\;v_{s}^{2}\right)}\right]}^{1/3}\approx A\;t\;{\left(\frac{E_{s}}{E_{p}}\right)}^{\frac{1}{3}}$$

where *λ* is the pattern wavelength, *A* is an arbitrary constant, *t* is the thickness of the top rigid layer, *E* is the Young modulus, *v* is the Poisson ratio, and the subindices *s* and *p* refer to skin and polymer bulk, respectively (i.e., to the rigid top layer and the soft foundation). In this case, the Poisson ratios of both (rigid skin and soft foundations) are taken as 0.5 because the hydrogel, formed by a high amount of water, is considered as an incompressible material. As can be concluded from Equation 1, the stiffness of the foundation is inversely proportional to wrinkle wavelength dimensions generated after plasma exposure. Interestingly, if the stiffness is higher enough to reach a critical value (14 h of vacuum), the surface does not deform after the external stimulus application (argon plasma exposure).

**Figure 6:**
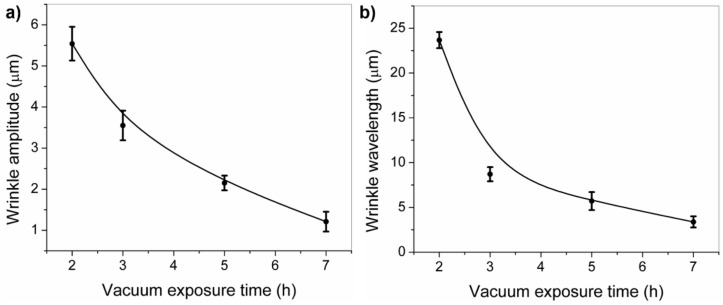
(**a**) Amplitude and (**b**) wavelength of the wrinkled patterns formed at different vacuum exposure times.

In Figure 6 are plotted the tendency of both the amplitude and the wavelength of the wrinkled patterns formed as a function of the vacuum exposure times. These results were obtained from a specially designed image analysis code based on MatLab [8,37], which measures automatically the width and the height of the wrinkles from a multi-level Otsu segmented image. The width of each wrinkle is obtained from the image Hough transformation (ellipse adjustment) and the height is estimated from a Gaussian single-peak fitting of the AFM micrograph profiles (Figure 5). As can be observed in both line plots, the vacuum exposure time considerably affects the wrinkle dimensions, either the amplitude or the wavelength. This effect makes it possible to alter the morphology of the patterns in an easy and reproducible way, just by changing the vacuum exposure time.

**Figure 7:**
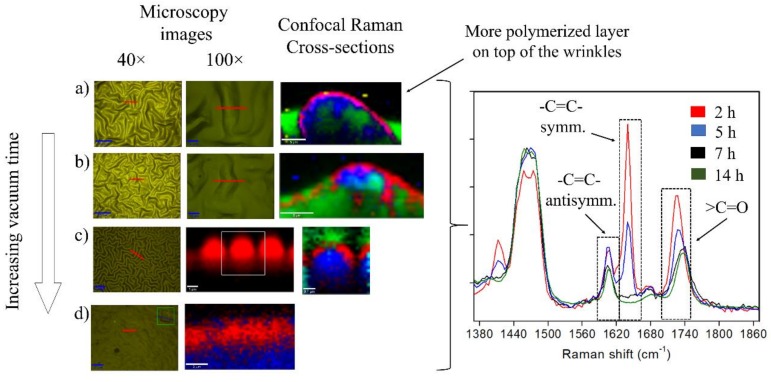
Optical images, confocal Raman cross-sections, and spectra for the samples prepared at increasing vacuum exposure time: (**a**) 2 h, (**b**) 5 h, (**c**) 7 h, and (**d**) 14 h.

Additionally, the formation of an oxidized layer on top of the material was corroborated by using confocal Raman spectroscopy. Moreover, the chemical effect that generated the vacuum exposure time on the films was detected by using this technique. Figure 7 shows some of the most relevant results of the confocal Raman spectroscopy performed on samples with different vacuum exposure times.

First, the analyses were focused on the evaluation of some chemical changes produced at different depths or strata of the film. From the Raman spectrum, we can estimate that when the samples were exposed to 2 h and 5 h of vacuum, one intense band located at 1647 cm^−1^ is observed. This signal is associated with -C=C- symmetrical stretching, commonly from the unreacted HEMA or PEGDA_575_ which remains after the thermal/photopolymerization. In the case of the sample prepared with 7 h and 14 h of vacuum, this signal completely disappears, indicating that the vacuum process over long periods probably facilitates the evaporation of the unreacted monomers from the film.

According to the Raman spectroscopy cross-section mapping, the unreacted monomers appear mainly in the valleys of the wrinkled patterns, probably due to a gravitational effect. Additionally, a thin layer on the top of the film was found in which the ratio of intensities between the bands 1607 cm^−1^ and 1737 cm^−1^ was considerably lower. The first peak is related to the antisymmetric stretching of the -C=C- bond, and the second with the >C=O stretching mode, which means that the top layer possesses more >C=O groups, probably due to oxidation generated during the plasma exposure process.

As mentioned before, the sample prepared with 14 h of vacuum did not present wrinkled patterns, which probably occurred because the sample was totally polymerized due to the evaporation of unreacted monomers, thus impeding the mechanical deformation of the top layer of the film. This effect was in part demonstrated in previous studies performed by our research group [8,37]. Additionally, it is important to mention that the signal ratio between the bands 1607 cm^−1^ and 1736 cm^−1^, as indicated below, was slightly higher compared with that of the samples prepared at 2 h, 5 h, and 7 h of vacuum. Moreover, a signal located at 1412 cm^−1^, which could correspond to the -CH_3_ bending and -CH_2_- symmetric scissoring modes, tended to disappear with the increase of vacuum exposure time. This band is characteristic of HEMA monomer according to González-Henríquez et al. [8]. Finally, for the sample obtained with 14 h of vacuum, one peak at 979 cm^−1^ was observed, which could be probably related to the in-phase wagging of >CH- group, to the -C-C- skeletal stretching, or to the symmetrical -CH_3_ rocking modes from the terminal carbons of the main chain. This signal is common from the *poly*(HEMA) chemical structure, indicating that unreacted HEMA almost disappears from the film due to evaporation.

### 2.3. Plasma Exposure Time Variation

**Figure 8:**
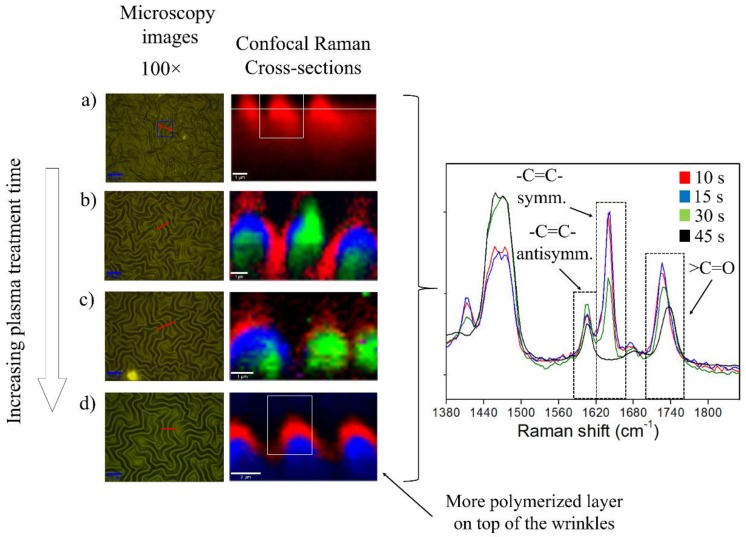
Optical images, confocal Raman cross-sections, and spectral analysis for samples fabricated at increasing plasma treatment time: (**a**) 10 s, (**b**) 15 s, (**c**) 30 s, and (**d**) 45 s.

Finally, in order to understand the effect produced by the argon plasma exposure on the material surface, samples with different exposure times were analyzed via confocal Raman spectroscopy and 3D optical profilometry. Raman spectroscopy results (Figure 8) show that the intensity ratio between the signals located at 1607 cm^−1^ and 1736 cm^−1^, which corresponds to the antisymmetric stretching of the -C=C- bond and to the >C=O stretching mode, respectively, were calculated using the sum of two Lorentzian adjusted curves. Four samples, which correspond to different argon plasma time exposures (10 s, 15 s, 30 s, and 45 s) were analyzed. For all the samples, the intensity ratio of the band located at 1736 cm^−1^ is greater on the surface than in the deeper strata of the film, indicating that a thin oxidized layer is formed on top of the samples. Interestingly, this ratio remains almost the same value for all the cases, indicating that an increase in plasma time exposure does not produce an over-oxidation of the surface. Apparently, the plasma time exposure increase generates a slight variation in the oxidized layer thickness, but it is not possible to categorically affirm it due to the low resolution of the confocal Raman spectroscopy in the cross-sectional direction.

Moreover, the results depicted above clearly indicate a lack of homogeneity in terms of chemical composition in the samples irradiated below 30 s. In fact, according to the confocal Raman experiment, in the samples fabricated after 10 s, 15 s, and 30 s of plasma exposure, it is possible to detect unreacted monomers (HEMA and PEGDA_575_) evidenced by the presence of a band located at 1645 cm^−1^, which is characteristic of -C=C- symmetrical stretching. In addition, in all the analyzed samples, a new vibrational mode appears at 1681 cm^–1^, which could correspond to the >C=O stretching mode of conjugated unsaturated aldehydes [38] or to the natural vibration of the -C=C-COOH group formed after argon plasma exposure [39,40].

**Figure 9:**
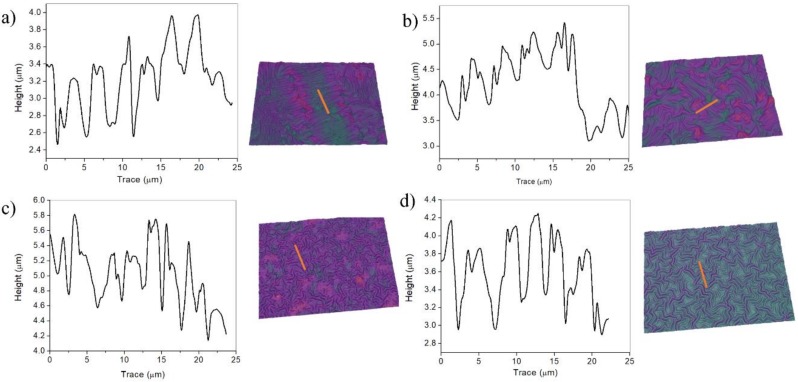
Height profiles and three-dimensional reconstructions obtained from optical profilometry for (**a**) 10 s, (**b**) 15 s, (**c**) 30 s, and (**d**) 45 s of argon plasma exposure time.

In contrast to this, samples irradiated for 45 s provide wrinkled surfaces with a clear thin rigid layer on top of a hydrogel substrate. The result of this transition can be easily observed in the 3D optical profiler images depicted in Figure 9. These images were used as a complementary analysis for the AFM micrographs. Profilometry allowed us to observe larger sectors of the sample, and to distinguish global orderings that were not detectable in the “local” domains measured by the AFM. On the one hand, Figure 9a–c indicates heterogeneous surfaces with wrinkles formed in domains, with preferential and inconstant directions and thus with variable characteristics of amplitude and period. On the other hand, Figure 9d clearly indicates the formation of a homogeneous wrinkled surface with a constant wrinkle size. In summary, it can be concluded that those samples, with a homogeneous chemical composition and a continuous thin rigid top-layer, also form homogeneous wrinkled surfaces.

## 3. Materials and Methods

### 3.1. Materials

All the solvents, monomers, and other reagents were used as received unless otherwise stated. The hydrogels used to form the films were synthesized using 2-hydroxyethyl methacrylate (HEMA, 97.0%) as main monomer and poly (ethylene glycol) diacrylate (PEGDA)—with an average molecular weight (M_n_) of 575 g mol^−1^—as a crosslinking agent. The photoinitiator used in this synthesis was 2-hydroxy-4′-(2-hydroxyethoxy)-2-methylpropiophenone (Irgacure 2959, 98.0%, Sigma-Aldrich, St. Louis, MO, USA). All these reactives were purchased from Sigma-Aldrich (St. Louis, MO, USA). A thermo-initiator, ammonium peroxodisulfate (APS, 98.0%, from Merck KGaA, Darmstadt, Germany), was employed with the purpose of initiating the gelation reaction and obtaining an appropriate viscosity for deposition.

Round glass coverslips (nominal thickness: 0.13–0.16 mm) from Ted Pella, Inc., (Redding, CA, USA) were employed as supports for depositing the hydrogel films. 1-Vinyl-2-pyrrolidone (NVP) stabilized with N,N′-di-sec-butyl-1,4-phenylenediamine, hydrogen peroxide (30–32%), sulfuric acid (95–97%) Emparta^®^ ACS, water for chromatography LiChrosolv^®^, and Glacial acetic acid (100%) Emsure^®^ ACS were obtained from Merck KGaA (Darmstadt, Germany). Finally, 3-(trimethoxysilyl) propyl methacrylate (TSM, 98.0%), from Sigma-Aldrich (St. Louis, MO, USA), was used to induce the substrate surface silanization with the purpose of improving the adhesion of the hydrogel films.

### 3.2. Methods

#### 3.2.1. Substrate Functionalization

First, the glass substrates were washed and sonicated for 5min in a solution of detergent and distilled water to remove any trace of surface contaminants, such as grease. Then, the cleaned substrates were submerged in a piranha solution (H_2_SO_4_:H_2_O_2_, 7:3) at 80 °C for 1 h [41]. This process generated hydroxyl groups (-OH) on the substrate surface, making it highly hydrophilic. Finally, the substrates were rinsed three times with MilliQ water and dried with a N_2__(g)_ flush (ultrapure).

The substrate functionalization was performed using a TSM solution (2% *v/v*) following the procedure reported by Yuk et al. [26]. For this purpose, a 100 mL portion of deionized water was acidified by adding glacial acetic acid drops until the solution reached a pH of 3.5. An aliquot of 40 mL was taken and mixed with 0.8 mL of N_2(g)_ purged TSM solution, which was then vigorously stirred for a few minutes. This solution was used to completely cover the substrate surface at atmospheric conditions for 2 h, enough time to allow methoxy silane molecules to chemisorb on the hydrophilic surface. Once the functionalization time was finished, the substrates were extensively rinsed with ethanol three times and dried with N_2(g)_.

In parallel, four different TSM solutions were prepared (0.005% *v/v*, 0.1% *v/v*, 1% *v/v*, and 2% *v/v*) following the methodology proposed by Yuk et al. [26]. This study was carried out to understand the TSM concentration influence that produced the self-assembled monolayer (SAM) on the hydrogel adhesion. It was expected that a major concentration of TSM in the substrate surface would produce a higher adhesion of the hydrogel film, thereby altering the wrinkled pattern morphology due to variations in the mechanical conditions during deswelling. The wrinkled pattern formed on top was characterized by AFM technique in each case; in addition, cross-hatch adhesion tests were performed for the different TSM concentration cases.

#### 3.2.2. Prepolymerization of the Hydrogel (Step 1)

The hydrogel synthesis was carried out in a glass vial and, accordingly, 1g of HEMA (monomer), 1g of PEGDA (crosslinking agent), and 10 mg of APS (thermo-initiator) were dissolved in 625 µL of deionized water. In parallel, 17.5 mg of Irgacure 2959 (photoinitiator) was dissolved in 100 µL of NVP in a microcentrifuge tube. This solution was homogenized and poured into the glass vial with the hydrogel mixture. Then, the vial was covered from light and purged with ultrapure N_2(g)_ in order to remove the remaining oxygen, which could generate undesired free-radical reactions. Finally, the vial was heated in a thermoregulated bath at 50 °C for 30min until reaching an adequate viscosity (80–120 cP) according to González-Henríquez et al. [8,37]. The solution viscosity was measured in situ via the methodology explained in those articles, generating a pre-polymerized hydrogel with a global conversion close to 50–60%.

#### 3.2.3. Sample Deposition (Step 2)

The doctor blade system corresponded to a home-made assembly which used a syringe pump (LSP01–1A/2A, Single Channel, Longer Precision Pump Co, Hebei, China) as a base. This equipment allowed us to move the substrate precisely and horizontally with a controlled speed and high spatial resolution (0.156 μm). An immobilized 90°-beveled razor blade was gently positioned over the substrate using a single axis translation stage from Edmund Optics Inc. (Barrington, NJ, USA). This assembly allowed us to move the razor blade a predetermined distance from the substrate surface. The thickness of the prepared film was directly correlated with this distance, the viscosity of the polymer, and the coating speed inserted in the syringe pump, among other ambient factors. The functionalized substrates were placed on the doctor blade mobile surface by fixing them with double contact adhesive tape, and the distance between the knife and the substrate was set at 10 µm. Then, an 8 µL portion of the oligomeric solution was placed on the substrate surface with a micropipette. The speed of the mobile surface was set at 0.2 mm/s in order to generate a thin hydrogel film homogeneously deposited on the substrate with high coverture.

#### 3.2.4. Vacuum Exposure and UV-vis Photopolymerization: Spontaneous Formation of Wrinkled Patterns (Step 3)

Once the films were successfully deposited using the doctor blade technique, the samples were exposed to vacuum during different periods (2, 3, 5, 7, and 14 h) in order to remove occluded solvent from the films. Interestingly, the material deswelling generated surface instabilities on the film due to the polymerization gradient produced, leading to shear stress mismatch between the unpolymerized and cured material layers. When the material was exposed to vacuum, the occluded water in the hydrogel was eliminated, which, upon an external stimulus, produced the release of accumulated stresses, triggering the formation of spontaneous wrinkled patterns on the hydrogel surface. The amount of water evaporated during this process could generate different wrinkle morphologies and sizes due to an increase or decrease in the stress released. The external stimulus applied to hydrogel films was, in this case, an Ar^+^ plasma exposure, which probably oxidized and polymerized the top layer of the film due to the generation of free radicals on the surface, that is, the combination of plasma and vacuum exposure triggered the spontaneous formation of wrinkled patterns. If hydrogel film is not exposed to plasma, the wrinkled pattern does not form, and a flat surface is obtained instead. An argon sputter coater (Cressington, 108 AUTO, Watford, UK) coupled with a high-resolution film thickness monitor (Cressington, MTM-20, Watford, UK) was used to generate Ar^+^ plasma etching on the hydrogel film surface.

**Figure 10:**
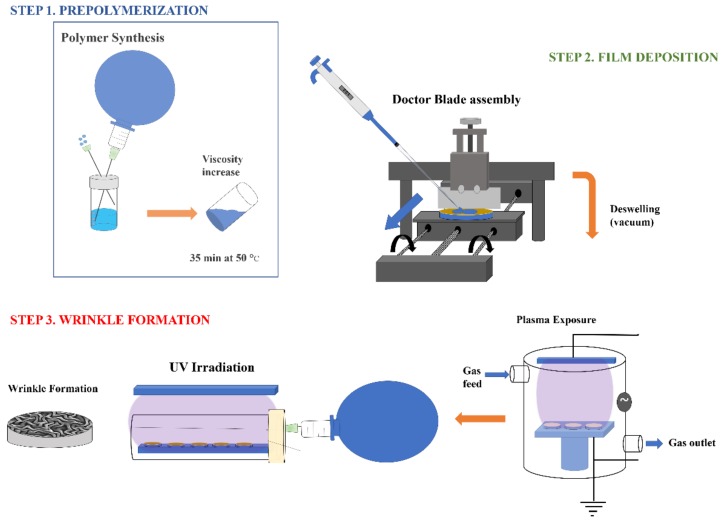
Schematic representation of the experimental procedure followed to form the spontaneous wrinkled patterns on top of the hydrogel films.

Finally, the sample was exposed to UV light in order to fully polymerize the film and fix the wrinkled pattern onto the hydrogel surface. UV-photo polymerizations were carried out through radiation exposure using a 9 W UV lamp with an emission peak centered at λ = 365 nm from Vilber Lourmat Inc. (Marne-La-Vallée, France). Figure 10 shows a schematic description of the methodology used in this study, starting with the synthesis and deposition until the wrinkled pattern formation via deswelling and plasma exposure processes.

By following the indications explained, the glass substrates were functionalized with TSM at a concentration of 2% (*v/v*) for 2h at room temperature [26]. Once the substrates were functionalized, the hydrogel films were deposited on top by using the doctor blade approach.

This methodology is highly innovative because it corresponds to a simple, cost affordable, and easily scalable method to deposit films [42]. It becomes important to mention that the monomeric mixture must be thermally polymerized (35 min at 50 °C) in order to obtain an appropriate solution viscosity to reach a homogeneous deposition.

The experimental settings employed for the preparation of the patterned surfaces play a major role in the wrinkle characteristics. The conditions and parameters used during the functionalization step, the duration of the vacuum exposure, and the plasma treatment were varied, providing an interesting methodology to finely tune the wrinkle dimensions as well as the homogeneity and the distribution of the surface patterns.

### 3.3. Characterization

The SAMs’ thicknesses for the four different TSM concentrations were obtained by ellipsometry. In addition, water contact angle studies over functionalized glasses were carried out.

Optical microscopy (OM) was used as a first approach to observing the morphology of the sample deposited over the TSM monolayer. A Bresser Trino Researcher II (40–1000×) trinocular microscope (Rhede, Germany), coupled with a CCD color camera (5 Mp, Bresser GmbH, Rhede, Germany) and with a cold light model CL-41 (OPTIKA©Srl, Ponteranica, Italy), was used for visualizing the topography of the hydrogel wrinkled patterns.

Additionally, the tridimensional topography of the samples was obtained via AFM using an AFM model NTEGRA Prima, from NT-MDT Co. (Moscow, Russia), in intermittent contact mode at different scan ranges (20 × 20 μm^2^ and 50 × 50 μm^2^). Force spectroscopy measurements were performed with a NaioAFM from Nanosurf Inc. (Woburn, MA, USA) using a tip specially designed for this purpose (PPP-FMR from NanoWorld AG, Liestal, Switzerland). For these studies, the sample was briefly imaged in AFM contact mode with the finality of identifying ridges and grooves in the surface topography. Subsequently, 12 different points were selected in order to obtain statistically reliable information. The results were then analyzed using the Hertz model for indentation with conical/pyramidal AFM tips over a soft flat surface [35,36]. Images were treated using the off-line software Gwyddion 2.42 Freeware (Brno, Czech Republic) [43].

After the analysis of this data, the height and width of the hydrogel wrinkled patterns were obtained using a MatLab code [37,42] specially designed for this purpose. In order to determine the relation between the TSM concentrations (0.005, 0.1, 1, and 2% (*v/v*)) and the adhesion force generated between the hydrogel deposited and the SAMs, cross-hatch adhesion tests were performed.

In parallel, confocal Raman spectroscopy studies were performed on the samples in order to corroborate the formation of an oxidized layer on the top of the material. The chemical composition and depth profiles of the polymeric films were determined using a CRM-Alpha 300 RA (WITec, Ulm, Germany) equipped with a Nd:YAG dye laser (maximum power output of 50 mW at 532 nm). The Raman spectra were taken point by point with a resolution step of 100 nm. Cross-section images were acquired using this methodology, and the relative intensities between different Raman signals were analyzed to form these images. Finally, a tridimensional representation of the surfaces was obtained using a profilometer, whose results allowed us to determine the roughness of the films, which were compared to the values obtained from AFM analysis. These cross-sectional profiles were obtained using a Zeta-20 optical profiler (Zeta Instruments, San Jose, CA, USA) with different optical objectives (5×, 20×, 50×, and 100×). The equipment has 13 nm in vertical resolution. The arithmetic average of the roughness absolute values (Ra) was obtained using the Zeta3D™ metrology systems (San Jose, CA, USA).

## 4. Conclusions

In this study, wrinkled patterns were formed on the surface of hydrogel films via the exposition of the samples to two different stimuli, namely, deswelling and surface oxidation. This was achieved via a two-step process, first a vacuum exposure to extract surface or occluded water from the samples followed by an argon plasma exposure step which produced surface oxidation via free-radical generation on the top of the material. To deposit the samples, the doctor blade technique was used, a methodology which is cost affordable and easily scalable, making it a very interesting solution for industrialization processes. This technique allows fabricating homogenous films with a controllable thickness and high surface coverage in short time periods.

The deposition parameters were tuned in order to generate a smooth and reproducible surface. Round glass coverslips were used as substrates, which were first functionalized with TSM solution with the purpose of enhancing surface adhesion between the hydrogel film and the substrate. This functionalization allowed the adhesion to increase from 8% to approximately 70%. These variations also produced changes on the pattern morphology due to a mechanical impediment that generated the adhesion increase, thus making it more difficult for the film to deform and therefore producing different patterns according to the functionalization degree of the substrate. Similarly, deswelling time also produced important changes in the wrinkled surface pattern morphology, varying the wrinkle width from ~24 µm to ~3.5 µm. In parallel, the chemical alteration of the hydrogel was also analyzed by using confocal Raman spectroscopy, a technique allowing us to demonstrate that a thin layer of oxidized hydrogel was formed on top due to plasma exposure.

## Figures and Tables

**Table 1:** Results of cross-hatch adhesion tests for hydrogel films deposited over silanized substrates at different TSM concentrations.

TSM Concentration (*v/v*)	Area Attached after Peeling
0.005%	5.3 ± 2%
0.1%	8.5 ± 3%
1%	25.0 ± 2%
2%	73.0 ± 3%

## Supplementary Materials

The Supplementary Materials are available online and include a figure with the amplitude and wavelength of the wrinkled patterns obtained at different TSM concentration.
