# Supplementary material for: Micrometric Wrinkled Patterns Spontaneously Formed on Hydrogel Thin Films via Argon Plasma Exposure
Source: Molecules. 2019 Feb 19;24(4):751. doi: 10.3390/molecules24040751 (PMC6412580; doi:10.3390/molecules24040751)
Supplement: Supplementary file 1 [file molecules-24-00751-s001.pdf]

Supplementary Materials

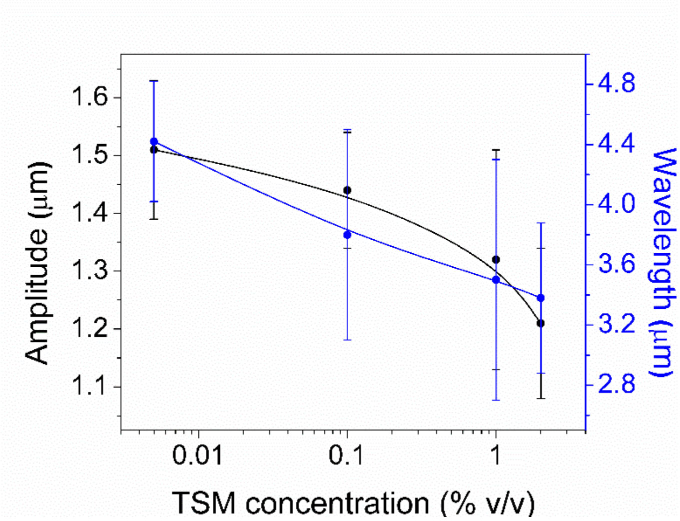

Figure S1. Amplitude and wavelength of the wrinkled patterns obtained by using different concentrations of TSM (0.005 %, 0.1 %, 1 %, and 2 % *v/v*) during functionalization process.
